# Measuring temporal bias in sequential numerosity comparison

**DOI:** 10.3758/s13428-024-02436-x

**Published:** 2024-05-15

**Authors:** Serena Dolfi, Alberto Testolin, Simone Cutini, Marco Zorzi

**Affiliations:** 1 Department of Developmental Psychology and Socialization, University of Padova, Via Venezia 8, 35131 Padova, Italy; 2 Department of General Psychology, University of Padova, Via Venezia 8, 35131 Padova, Italy; 3 Department of Mathematics, University of Padova, Padova, Italy; 4 IRCCS San Camillo Hospital, Venice, Italy

**Keywords:** Numerical cognition, Continuous magnitudes, Temporal numerosity, Duration, Vision, Audition

## Abstract

While several methods have been proposed to assess the influence of continuous visual cues in parallel numerosity estimation, the impact of temporal magnitudes on sequential numerosity judgments has been largely ignored. To overcome this issue, we extend a recently proposed framework that makes it possible to separate the contribution of numerical and non-numerical information in numerosity comparison by introducing a novel stimulus space designed for sequential tasks. Our method systematically varies the temporal magnitudes embedded into event sequences through the orthogonal manipulation of numerosity and two latent factors, which we designate as “duration” and “temporal spacing”. This allows us to measure the contribution of finer-grained temporal features on numerosity judgments in several sensory modalities. We validate the proposed method on two different experiments in both visual and auditory modalities: results show that adult participants discriminated sequences primarily by relying on numerosity, with similar acuity in the visual and auditory modality. However, participants were similarly influenced by non-numerical cues, such as the total duration of the stimuli, suggesting that temporal cues can significantly bias numerical processing. Our findings highlight the need to carefully consider the continuous properties of numerical stimuli in a sequential mode of presentation as well, with particular relevance in multimodal and cross-modal investigations. We provide the complete code for creating sequential stimuli and analyzing participants’ responses.

**Supplementaryinformation:**

The online version contains supplementary material available at 10.3758/s13428-024-02436-x.

Humans can rapidly estimate the number of elements in a collection of items without counting, although in an imprecise manner. Sensitivity to numerical information has been observed even in young infants (Xu & Spelke, 2000) and several nonhuman species (e.g., Agrillo et al., 2008; Bortot et al., 2019; Cantlon & Brannon, 2007), and can emerge from statistical learning mechanisms in deep neural networks (Stoianov & Zorzi, 2012; Zorzi & Testolin, 2018; Testolin, Zou & McClelland, 2020b). Nonsymbolic number processing is thought to be independent of mode of presentation (e.g., arrays of items vs. sequences of flashes) or sensory modality (e.g., visual vs. auditory), since individuals show similar precision in discriminating numerosity from visual collections of elements and sequences of visual, auditory, or tactile events (Anobile et al., 2018; Barth et al., 2003; Tokita & Ishiguchi, 2016). Moreover, the ability to integrate numerical information across sensory modalities seems to appear early in life (Izard et al., 2009; Jordan & Brannon, 2006), and a supra-modal encoding of numerical information is further supported by adaptation studies (Arrighi et al., 2014) and neuroscientific findings (Eger et al., 2003; Nieder, 2012). However, inconsistencies between sensory modalities and mode of presentation have been reported (Droit-Volet et al., 2008; Tokita et al., 2013), including at the neural level (Cavdaroglu & Knops, 2019), calling for the design of more precise experimental paradigms to control for potential confounds.

A long-debated methodological issue in the study of numerosity perception is the possible influence of continuous magnitudes on numerosity judgments. Indeed, converging evidence shows that visual numerosity judgments are influenced by non-numerical quantities such as total surface area, convex hull, or density (Clayton et al., 2015; Dakin et al., 2011; Gebuis & Reynvoet, 2012). According to one hypothesis, these interactions can be explained by a partially overlapping representation of numerical and non-numerical quantities (Walsh, 2003), subserved by shared neural resources (Sokolowski et al., 2017, for a meta-analysis). Others postulate an interference between competing representations at the response selection level after initial parallel processing (Gilmore et al., 2013; Nys & Content, 2012), as also suggested by the variability in interference effects across different contexts (Dramkin et al., 2022). An alternative view instead argues that the influence of non-numerical visual cues suggests that numerical information might be indirectly extracted by weighting non-numerical quantitative information, thus calling into question the very concept of “number sense” (Gebuis et al., 2016; Gevers et al., 2016). Despite the lack of consensus on the underlying mechanism, the tight interplay between continuous visual magnitudes and numerosity is well documented, and several methods have been suggested to assess and estimate the presence of non-numerical biases in parallel numerosity comparison tasks (DeWind et al., 2015; Salti et al., 2017) and to practically generate numerical stimuli with a precise manipulation of their visual features (De Marco & Cutini, 2020).

Nevertheless, the possible effect of continuous temporal cues on sequential numerosity perception is still largely unexplored. A few studies have reported significant interference from temporal duration during parallel numerosity comparison, with an overall overestimation of arrays that are displayed for a longer duration (Javadi & Aichelburg, 2012). However, the latter finding cannot be disentangled from the possible effect of exposure duration (Inglis & Gilmore, 2013). Investigations based on a sequential or dynamic presentation of events offer a contradictory picture. Some studies indicate that duration can influence sequential numerosity judgments, but with divergent results regarding the direction of its effect (Lambrechts et al., 2013; Martin et al., 2017; Philippi et al., 2008; Togoli, Fornaciai, et al., 2021b; Tokita & Ishiguchi, 2011). On the other hand, a large portion of studies on developmental or adult populations failed to find any influence of the duration of the stimuli on numerical judgments of sequences of visual or auditory events (Agrillo et al., 2010; Dormal et al., 2006; Dormal & Pesenti, 2013; Droit-Volet et al., 2003). Such inconsistencies can be partially explained by the lack of a common methodology to investigate temporal biases, and by the fact that most studies limited their investigations to the effect of either the duration of the overall sequence/stimulus or the intervals between events, ignoring potential interferences related to other covarying temporal dimensions such as the duration of individual events.

To overcome this issue and enable more precise investigations on the nature of numerical representation, considering its relationship to temporal magnitude processing, here we extend to the temporal domain a recent framework used to model responses in parallel numerosity comparison tasks (DeWind et al., 2015; Park, 2022). Our goal is to provide a common ground for the investigation of biases generated by continuous magnitudes on parallel and sequential numerosity perception, at the same time promoting the investigation of the interplay between numerical and non-numerical magnitude processing in different sensory modalities. We first describe the method for generating sequences of events that vary in number, duration, and distance in time, as well as the statistical method for response modeling (MATLAB code is made available online^1^). To demonstrate its applicability in numerosity perception studies, we then validate the proposed method in two psychophysical experiments involving both visual and auditory modalities, where participants performed a numerosity comparison task with sequences of either flashing dots or tones.

## Proposed method

The framework originally developed in the seminal work of DeWind and colleagues (2015; also see Park, 2022, for discussion) consists of the systematic construction of nonsymbolic numerical stimuli by varying several continuous magnitudes alongside numerosity, taking into account the mathematical relationships between numerical and non-numerical features. We extended the original idea to sequences of events, which are characterized not only by their number but also by temporal features.

**Fig. 1:**
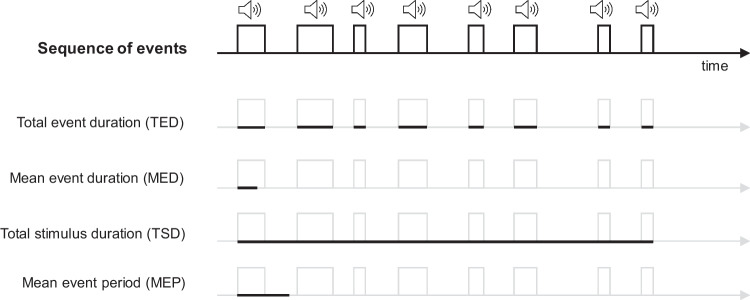
Temporal magnitudes in sequential non-numerical stimuli. Schematic representation of an example of an auditory sequence of events. Black sections of the sequence timeline highlight on separate rows the temporal features considered by the illustrated method.

In particular, we distinguish between intrinsic temporal features, which pertain to the individual events, and extrinsic temporal features, which are related to the entire sequence (see Fig. 1). Following DeWind et al. (2015), we define mean event duration (MED) as the average duration of the individual events and total event duration (TED) as the sum of the length of all pulses (similar to average item size and total surface area, respectively, in spatial arrays of elements). In the case of regular sequences with fixed durations of events, mean event duration corresponds to individual event duration (IED) (equal to individual item size)*.* Similarly, we refer to total stimulus duration (TSD) as the time from the beginning of the first pulse to the end of the last pulse, intervals included, and mean event period (MEP) as the total stimulus duration divided by the number of events (similar to convex hull and sparsity).

**Fig. 2:**
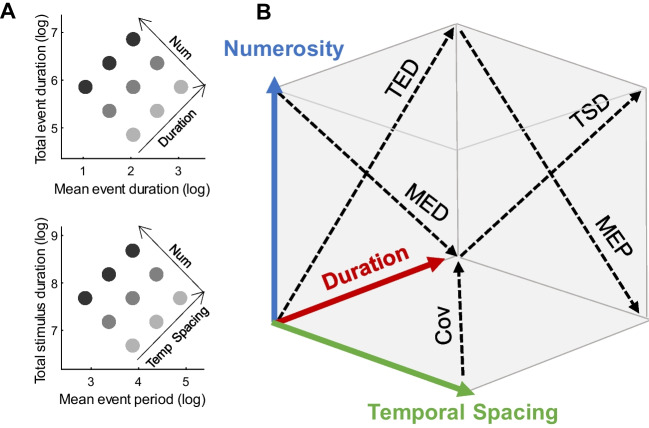
Relationship between extrinsic and intrinsic temporal features. **A** Depiction of the relationship between numerosity and the considered intrinsic and extrinsic temporal magnitudes. Duration is the axis orthogonal to numerosity representing a change in total event duration (TED) and mean event duration (MED). Similarly, temporal spacing is the axis representing a change in both total stimulus duration (TSD) and mean event period (MEP). **B** Schematic representation of the three-dimensional parameter space defined by taking numerosity, duration, and temporal spacing as cardinal axes.

Based on the relationship between intrinsic and extrinsic features, we can derive two dimensions orthogonal to numerosity, namely duration and temporal spacing (see Fig. 2a and b). In analogy with the visual features introduced by DeWind and colleagues (2015), these two dimensions can be mathematically defined as

$$\begin{array}{c}Duration=TED\times IED\\ Temporal \;Spacing=TSD\times MEP\end{array}$$

or, in logarithmic scale:

$$\begin{array}{c}{log}_{2}\left(Duration\right)= {log}_{2}\left( TED\right)+{log}_{2}\left( MED\right)\\ {log}_{2}\left(Temporal \;Spacing\right)= {log}_{2}\left( TSD\right)+{log}_{2}\left({\text{MEP}}\right)\end{array}$$

In this sense, duration is the dimension that varies simultaneously with TED and MED, keeping numerosity constant, while temporal spacing is the dimension that varies simultaneously with TSD and MEP, keeping numerosity constant. For a fixed number of events, a change in duration is associated with a change in the average temporal length of the events spread in a fixed interval, while a change in temporal spacing can be imagined as a change in their temporal distance, keeping a fixed average duration.

Conversely, we can mathematically describe the individual temporal features in terms of the three cardinal dimensions numerosity (*n*), duration (Dur), and temporal spacing (TmSp) as

$$\begin{array}{c}TED=\sqrt{Dur \times n}\\ MED=\sqrt{Dur/n}\\ \begin{array}{c}TSD=\sqrt{TmSp \times n}\\ MEP=\sqrt{TmSp/n}\end{array}\end{array}$$

In addition to the features described above, we can consider coverage (Cov) as an alternative measure of density, as total event duration per stimulus duration:

$$Coverage=\sqrt{Dur/TmSp}$$

Note that log scaling the axes produces a linear relationship between the cardinal dimensions and the other temporal features (see Table 1). Moreover, in log space, the distance between stimulus points becomes proportional to the ratios of their features.

**Table 1:** Algebraic relationship between several temporal features and cardinal features in log space

	Log of feature in terms of log of three orthogonal dimensions	Log of feature ratio in terms of log of three orthogonal ratios
Total event duration (TED)	${\text{log}}\left(TED\right)= \frac{1}{2}{\text{log}}\left(Dur\right)+ \frac{1}{2}{\text{log}}\left(n\right)$	${\text{log}}\left({r}_{TED}\right)= \frac{1}{2}{\text{log}}\left({r}_{Dur}\right)+ \frac{1}{2}{\text{log}}\left({r}_{n}\right)$
Mean event duration (MED)	${\text{log}}\left(MED\right)= \frac{1}{2}{\text{log}}\left(Dur\right)- \frac{1}{2}{\text{log}}\left(n\right)$	${\text{log}}\left({r}_{MED}\right)= \frac{1}{2}{\text{log}}\left({r}_{Dur}\right)- \frac{1}{2}{\text{log}}\left({r}_{n}\right)$
Total stimulus duration (TSD)	${\text{log}}(TSD)= \frac{1}{2}{\text{log}}\left(TmSp\right)+ \frac{1}{2}{\text{log}}(n)$	${\text{log}}({r}_{TSD})= \frac{1}{2}{\text{log}}\left({r}_{TmSp}\right)+ \frac{1}{2}{\text{log}}({r}_{n})$
Mean event period (MEP)	${\text{log}}(MEP)= \frac{1}{2}{\text{log}}\left(TmSp\right)- \frac{1}{2}{\text{log}}(n)$	${\text{log}}({r}_{MEP})= \frac{1}{2}{\text{log}}\left({r}_{TmSp}\right)- \frac{1}{2}{\text{log}}({r}_{n})$
Coverage (Cov)	${\text{log}}(Cov)= \frac{1}{2}{\text{log}}\left(Dur\right)- \frac{1}{2}{\text{log}}(TmSp)$	${\text{log}}({r}_{Cov})= \frac{1}{2}{\text{log}}\left({r}_{Dur}\right)- \frac{1}{2}{\text{log}}({r}_{TmSp})$

In our online repository, we provide a working example of code that can be used to create a stimulus set taking into consideration the features described above. Stimuli are first defined as sequences of timestamps in frames, in order to easily instantiate either visual or auditory sequences with identical temporal properties through different stimulus presentation software. Users can generate regular (with fixed IED and fixed inter-event intervals) or irregular sequences. From the script, users can also easily visualize the relationship between numerosity and the mentioned non-numerical features in the resulting sequential numerical stimuli. A thorough description of the code is provided in the Supplementary Material.

Stimuli generated by orthogonally varying numerosity, duration, and temporal spacing can then be used to estimate the impact of non-numerical magnitudes on the dependent variable using a regression-based approach. Approximate numerical representations have been traditionally modeled as a logarithmically compressed number line, where numerosities are represented by partially overlapping Gaussian distributions in accordance with Weber’s law (Dehaene, 2003; Piazza et al., 2004), so that the discriminability of a change in numerosity is dependent on the difference in log numerosity. Similarly to DeWind et al. (2015), we can extend the same logic to non-numerical magnitudes and estimate how performance in behavioral tasks is affected by log differences in temporal cues. For example, in a sequential numerosity comparison task, trial-by-trial responses can be modeled with a generalized linear model using as regressors the log-ratios of numerosity, duration, and temporal spacing between the two sequences. The combination of the estimated coefficients of the regressors (β_Num_, β_Dur_, and β_TempSp_) is informative regarding the influence of numerical and non-numerical quantities on participant’s decisions. If the response is based entirely on numerical ratios and is unaffected by non-numerical temporal features, it will lead to a positive coefficient for numerosity and coefficients for duration and temporal spacing close to zero. In this context, β_Num_ can also be considered an indication of numerical acuity, with larger values of the numerosity coefficients corresponding to better performance in discriminating more difficult ratios (De Wind et al., 2015). Nonzero values for the β_Dur_ and β_TempSp_ coefficients instead quantify the influence of temporal features on the participant responses.

The three coefficients can also be thought of as defining a discrimination vector in the parameter space: the vector norm depends on the overall discrimination acuity, while its orientation is informative about the relevant features determining performance (DeWind et al., 2015). In the case of a response unbiased by temporal magnitudes, the discrimination vector is perfectly aligned with the numerosity axis. Significant non-numerical biases, instead, cause the vector to deviate from the numerosity dimension; in such a case, its angle from the numerosity axis can be used to quantify non-numerical bias irrespective of the orientation of the discrimination vector. Moreover, as extensively described by Park (2022), the linear relation (see the equations in Table 1) between the cardinal dimensions of this stimulus space and individual features allows one to derive the impact of individual features on participants’ response from the combination of parameters estimated for the cardinal axes, while avoiding multicollinearity between regressors. For example, a participant consistently selecting the sequence with a longer total duration of the events would be characterized by a positive and equal coefficient for numerosity and duration.

The proposed extension of the framework introduced by DeWind and colleagues (2015) to the temporal domain thus enables a precise quantification of the contribution of temporal features, allowing us to the estimate the overall non-numerical bias on task performance as well as the test of specific hypotheses regarding individual temporal features potentially influencing or driving task response.

### Validation: Psychophysical experiments

To evaluate the proposed method and sequential stimuli, we conducted two online experiments where participants performed a numerosity comparison task in either visual or auditory modalities. The objectives of the study were twofold. On one hand, we aimed to empirically evaluate the methodology and test its versatility. At the same time, we sought to better characterize, in the healthy adult population, the effect of temporal magnitudes on numerosity judgments and identify potential differences between sensory modalities in strategy, presence of non-numerical bias, and numerical acuity.

#### Method and materials

##### Participants

One hundred and forty individuals took part in the study, with 69 participants completing the comparison task in the visual modality and 71 in the auditory modality. Due to the internet-based data collection procedure (see below), we excluded 13 participants for technical issues related to the presentation of the stimuli (e.g., incorrect screen refresh rate) and 28 participants for showing poor comprehension of task instructions (accuracy below 50% in practice trials) or low attention during the task (low accuracy in the easiest trials or a high number of outlier response times). Finally, 13 additional participants were excluded during the analyses due to a non-satisfactory fit of the statistical model (see Data Analysis section for details on all exclusion criteria). The final sample was composed of 41 participants (28 females) with a mean age of 24.12 (range: 20 – 36) for the visual modality and 45 participants (34 females) with a mean age of 22.47 (range: 20 – 31) for the auditory modality. A power analysis conducted using Monte Carlo simulations (see Supplementary Material) indicated a minimum sample size of 20 participants to detect a non-numerical bias with power above .90, a minimum sample of 20 participants per group to detect a group difference in non-numerical bias with power above .90, and a minimum sample of 40 participants per group to detect a group difference in numerical acuity of *d* = 1 with power above .70. Participants were volunteers recruited through social media and students from the University of Padova who received course credits for their participation. All participants gave their written informed consent. Research procedures were approved by the Psychological Science Ethics Committee of the University of Padova.

##### Procedure

The experiment was conducted online through Pavlovia, a hosting platform for running online PsychoPy tasks (www.pavlovia.org). Participants were instructed to perform the task in a single session, on a laptop or computer, in a quiet environment without distractions, sitting approximately one arm from the screen. In the case of the auditory comparison task, participants were free to use the speakers or headphones connected via cable to the computer; 18 participants performed the task using a headset or earphones, while the remaining ones used the computer speakers.

##### Stimuli

We varied independently numerosity, duration, and temporal spacing across 13 levels evenly spaced on a logarithmic scale. Numerosity varied between 7 and 28, and a similar maximum range of 1:4 was used for duration and temporal spacing. Sequences were not homogeneous, so the individual duration of an event and the single interval between one pulse and the next could vary within the same stimulus, with events lasting between 2 and 16 frames (33–270 ms at 60 Hz) and empty intervals lasting between 3 and 30 frames (50–500 ms). Such minimum durations are considered to be above the visual and auditory temporal resolution thresholds (Kanabus et al., 2002). An initial dataset of 500 pairs was created; to build each pair, we selected a ratio between 1.1:1 and 2:1 independently for numerosity, duration, and temporal spacing. Then sequences were created from the combination of appropriate pairs of numerosity, duration, and temporal spacing randomly selected from the 13 levels. For each participant, at the beginning of the experiment, a random subsample of 120 stimuli was drawn from the initial dataset to obtain an equal range for the three orthogonal dimensions and a balanced distribution of the ratios in the entire dataset and presented in a randomized order.

Stimuli were created in MATLAB R2020a (www.mathworks.com) as sequences of timestamps and instantiated as sequences of visual or auditory events directly in PsychoPy/PsychoJS (Peirce et al., 2019), which enables online experiments to be run with accurate temporal precision (Bridges et al., 2020). Visual stimuli were presented as sequences of flashes (white discs on a gray background) placed centrally on the screen. The dimension of the dot scaled with the resolution of the participants’ screen. Auditory stimuli were presented as sequences of sounds (pure tones at 400 Hz). At the beginning of the experiment, participants were allowed to adjust the volume as they preferred. Independently from modality, all durations were controlled in frames.

##### Task

All participants performed a computerized numerosity comparison task with either visual or auditory sequences of events. They were presented with pairs of sequences of rapid flashes or tones and were instructed to indicate which one contained more events by pressing the left or right arrow keys to select the first or the second sequence, respectively. Each trial began with a fixation cross lasting 1 s, after which the two sequences were presented, one after the other, with a fixed interval of 2 s between the first and the second one. The duration of the sequences changed depending on the stimulus, ranging from 1.70 s to 6.83 s. To prevent participants from consistently overestimating the duration of the first stimulus, during the interval between the two sequences, a green fixation cross appeared in the center of the screen to indicate the end of the first sequence. After the second sequence, a blank screen was presented until the participant response, followed by a pseudorandom blank inter-trial interval between 500 and 1500 ms. Participants did not receive any feedback based on their responses. Both tasks consisted of 120 test trials (around 40 minutes), divided into four blocks; participants could take a short break between blocks. Before the test phase, each participant completed five easy practice trials (randomly selected from a pool of 30 pairs) with a numerical ratio equal to 1:4 (i.e., 7 vs. 28) and the ratios of non-numerical dimensions varying between 1.1:1 and 2:1. In the practice phase, we selected pairs with an easy numerical ratio and variable temporal features to let participants familiarize themselves with the task, making sure they understood the instructions and to prepare them for the variability in the temporal dimensions that they would experience in the test phase. During the practice phase, the trial structure was identical to that of the test phase, with the only difference that participants received feedback indicating whether their response was correct or not.

##### Data analysis

Differences in screen refresh rate between participants were examined a posteriori, and all participants with a refresh rate different from 60 Hz were discarded, independently from stimulus presentation modality. Participants who were not appropriately engaged in the task were excluded from the analysis. One participant in the visual task and two participants in the auditory task were discarded due to inaccurate performance in practice trials (below 50%). Furthermore, we considered the largest numerical ratio (1:2) as catch trials, discarding participants with an accuracy below 75% in this easiest condition. This led to the exclusion of 12 participants in each sensory modality. To remove unattended trials, in both modalities we also discarded trials where a response was recorded before 200 ms (anticipations) or later than 4 s (Halberda et al., 2012), planning to exclude participants if more than 20% of their total trials were discarded. Based on this cut-off, we excluded one additional participant in the auditory task.

We then modeled individual responses (selection of the second sequence) with a generalized linear model (GLM) with binomial distribution and probit link function, using as regressors the log-ratios (second/first sequence) of numerosity, duration, and temporal spacing. We excluded from subsequent analyses eight participants for the visual task and five participants for the auditory task whose performance was not well captured by the GLM, indicated by an *R*^2^_adj_ below 0.2 (Piazza et al., 2010). We tested the significance of coefficients at the group level with one-sample Student *t*-tests against zero. A positive coefficient for duration is associated with an overestimation of long events, while a negative coefficient indicates that shorter events are perceived as more numerous. Similarly, a positive coefficient for temporal spacing indicates that events separated by longer intervals are perceived as more numerous, while a negative coefficient is related to an overestimation of higher rates of events. The estimated coefficients were also used to calculate the nondirectional angle between individual discrimination vectors and the numerosity axis. To assess the proximity of the discrimination vector to the different feature dimensions, we computed the vector projections onto the dimensions of the temporal features, and we determined the closest to the discrimination vector at the group level with a series of paired *t*-tests. Nonparametric tests (Wilcoxon signed rank) were performed in case of violation of the normality assumption, and Bonferroni's method was used to correct multiple comparisons whenever necessary. We also assessed differences in acuity and bias between modalities with frequentist and Bayesian *t*-tests. In the latter case, we report the Bayes factor *BF*_10_, expressing the probability of current data under the alternative hypothesis (H1) relative to the null hypothesis (H0) (Kass & Raftery, 1995). Values larger than 1 are in favor of H1, and values smaller than 1 are in support of H0. A BF between 1 and 3 (or between 1 and 0.33) can be considered anecdotal evidence, a BF between 3 and 10 (0.33–0.10) can be considered moderate evidence, and a BF larger than 10 (< 0.03) can be considered as strong evidence (van Doorn et al., 2020). Bayesian analyses were conducted using JASP ver. 0.12.1 2020 (www.jasp-stats.org), with default priors. Analyses were otherwise performed with MATLAB.

## Results

The proportion of correct responses over the total number of trials was above chance in both the visual (mean 0.80, range 0.68–0.88) and auditory modality (mean 0.81, range 0.65–0.93). Mean response times ranged between 457 and 1498 ms (mean 878 ms) in the visual modality and between 454 and 1506 ms (mean 1010 ms) in the auditory modality.

**Fig. 3:**
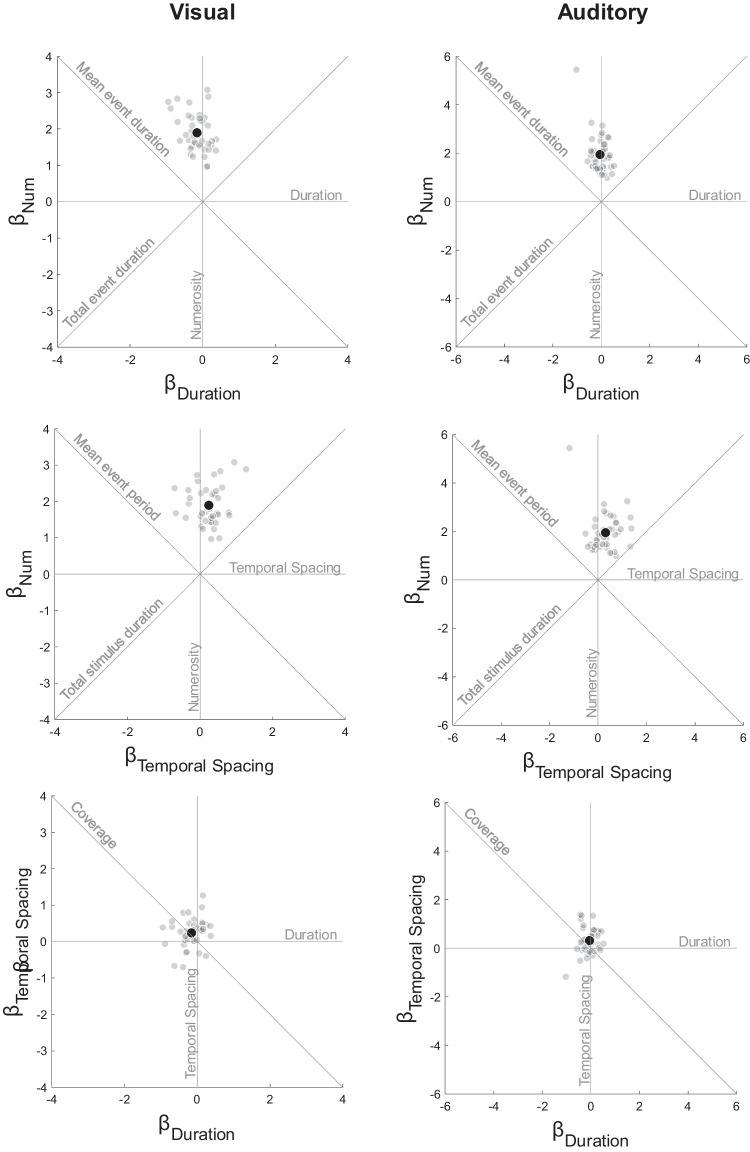
Results from GLM analysis of visual and auditory comparison tasks. Individual coefficient estimates are plotted in the three orthogonal planes defined by the cardinal axes, with visual modality in the left column and auditory modality in the right column. Gray dots indicate individual participants, while the black dots indicate the group means. Gray lines represent the temporal features.

**Fig. 4:**
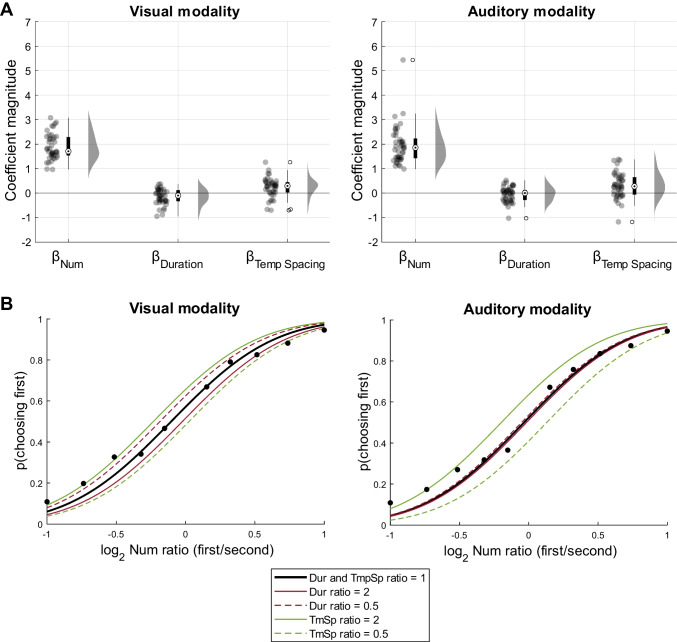
Group results of visual and auditory comparison tasks. **A** Distributions of individual coefficient estimates for numerosity, duration, and temporal spacing in the visual (left) and auditory modality (right). **B** Psychometric curves derived from the coefficients estimated at the group level. Black lines represent the predicted probability of choosing the first sequence as a function of the logarithm of the first to second numerosity ratios when the ratios of duration and temporal spacing are equal to 1. Red lines represent the same probability in trials with large ratios of duration and temporal spacing ratio equal to 1, while green lines are the predicted curves for trials with large temporal spacing ratios and duration ratio equal to 1. In both cases, full lines represent trials where the first sequence has a larger duration or temporal spacing than the second, and dashed lines indicate the opposite.

The GLM fit was significantly better than a constant model for all participants in the final sample of both the visual (mean *R*^2^_adj_ = 0.44, all χ^2^ > 29.82, all *p*s < .001) and the auditory (mean *R*^2^_adj_ = 0.46, all χ^2^ > 27.71, all *p*s < .001) tasks. The individual coefficient estimates are presented in Fig. 3, with additional lines representing non-orthogonal temporal features. At the group level, the model coefficients were significantly different from zero for numerosity (*M* = 1.89, *SD* = 0.53, *t*(40) = 22.96, *p* < .001, *d* = 3.59), duration (*M* = −0.16, SD = 0.32, *t*(40) = −3.21, *p* = .002, *d* = −0.50), and temporal spacing (*M* = 0.24, *SD* = 0.40, *t*(40) = 3.85, *p* < .001, *d* = 0.60) in the visual modality. For the auditory task, β_Num_ (*M* = 1.95, *SD* = 0.77, *t*(44) = 16.90, *p* < .001, *d* = 2.52) and β_TmSp_ (*M* = 0.31, *SD* = 0.51, *t*(44) = 4.06, *p* < .001, *d* = 0.61) were significantly different from zero at group level, while the coefficient magnitude of duration was not (*M* = −0.05, *SD* = 0.31, *t*(44) = −1.16, *p* = .25, *d* = −0.17) (see Fig. 4a).

For a better visualization of group results, we fit mixed-effect models separately for the two modalities, with similar fixed effects but including by-subject random intercepts and slopes of numerosity, duration, and temporal spacing. Psychometric curves obtained by the estimated fixed effect from these two models are presented in Fig. 4b. Both the individual results and the group model (further described in the Supplementary Material) indicate that our participants discriminated sequences primarily on the basis of the number of flashes and tones, but that they were also marginally affected by non-numerical temporal cues of the stimulus sequences.

**Fig. 5:**
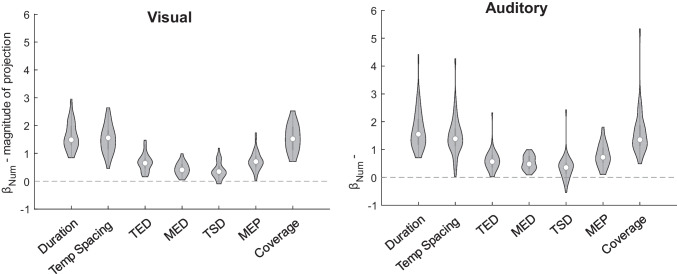
Results from projection analysis in visual and auditory tasks. Distribution of differences between the numerosity coefficient and the projection of the individual discrimination vector onto each temporal feature line for the visual (left column) and auditory modality (right column). Positive values indicate discrimination vectors closer to the numerosity dimension compared to the considered feature, while negative values indicate closer proximity to the specific feature.

The combination of coefficient weights suggest that participants’ responses were primarily based on a numerical strategy. This conclusion is further supported by the analysis of the discrimination vectors: we computed the vector projections onto the non-orthogonal dimensions at the individual level, and tested whether any other magnitude projection was higher than the numerosity coefficient. Differences between the numerosity coefficient and the magnitude projections are shown in Fig. 5. β_Num_ was higher than all the other projections on the total event duration, mean event duration, total stimulus duration, mean event period, and coverage dimensions in both the visual modality (paired *t*-tests with Bonferroni correction: all *t*s(40) > 8.16, *p*s < .01, *d*s > 1.28) and the auditory modality (Wilcoxon signed-rank tests with Bonferroni correction: all *W* > 930.00, *p*s < .01, *r* > 0.79).

**Fig. 6:**
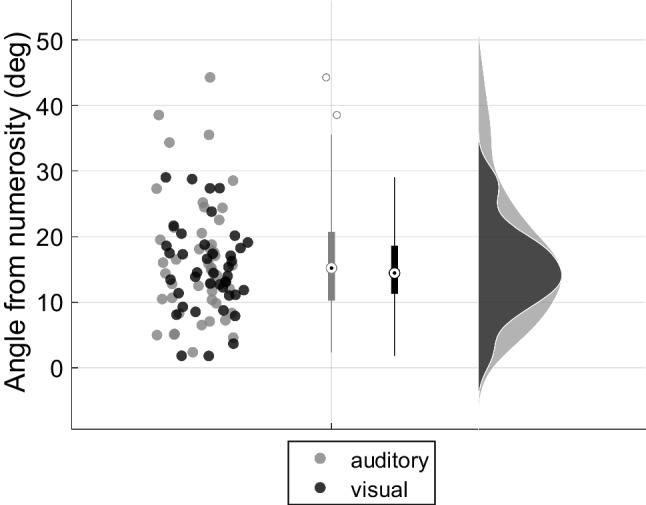
Comparison of vector line angle between modalities. Distribution of the angles (in degrees) between the individual discrimination vectors and the numerosity dimension in the two modalities (visual: black; auditory: gray)

The magnitude of the numerosity coefficient was not significantly different between the visual and auditory tasks (*t*(84) = 0.38, *p* = .70, *d* = 0.08, *BF*_*10*_ = 0.24 (0.03), moderate evidence), suggesting a similar numerical acuity across sensory modalities. As for non-numerical biases, we estimated the angle between the numerosity dimension and individual discrimination vectors as a nondirectional measure of non-numerical bias (see Fig. 6). No difference emerged in the vector line angle estimated in visual (*M* = 15.15°, *SD* = 6.59) and auditory (*M* = 16.50°, *SD* = 9.40) modality (*U* = 941, *p* = .88, *r = 0.02*, *BF*_10_ = 0.29 (0.03), moderate evidence). We directly compared the coefficients of duration between tasks, and we did not find a significant difference between the weights of duration in the two modalities (*t*(84) = 1.58, *p* = .12, d = 0.34, *BF*_10_ = 0.67), despite the significant result from the one-sample *t*-test in the visual modality.

## Discussion

The present work aimed to extend the methodology proposed by DeWind and colleagues (2015) to enable the assessment of temporal biases in sequential numerosity perception. Our main goal was to create a common framework for the study of both visual cues and temporal biases, facilitating investigations across multiple sensory modalities and modes of presentation, at the same time improving the comparability of measures used in the investigation of non-symbolic number processing. Besides describing the theoretical bases for the creation of the proposed stimulus space, we also provide computer code to generate sequences of timestamps where numerosity, duration and temporal spacing are orthogonally varied. We validated our method in an empirical study, demonstrating that it can be effectively used to quantify how much participants rely on numerical and temporal information during sequential numerosity discrimination, and to identify relevant cues impacting numerosity judgments in different sensory modalities.

Our findings show that adult individuals discriminated sequences primarily on the basis of the number of flashes and tones and were only marginally affected by non-numerical temporal cues embedded in the stimulus sequences. The prominent numerical strategy adopted by our participants adds to the growing body of evidence suggesting that performance in numerosity estimation tasks cannot be explained by the unique processing of non-numerical magnitudes (Abalo-Rodríguez et al., 2022; Cicchini et al., 2019; DeWind et al., 2015). However, although our analyses rule out the possibility that participants relied consistently on any single time cue considered, our behavioral investigation cannot exclude a more complex and dynamic integration of non-numerical magnitudes (Gebuis & Reynvoet, 2012). To address this issue, the stimulus space introduced in this work could be exploited in electrophysiological and neuroimaging studies, as done with the original space from which we took inspiration (DeWind et al., 2018; Fornaciai et al., 2017; but see Park, 2018). Furthermore, it would be interesting to adopt our sequential stimulus space to investigate whether numerosity information would still play a significant role even when the task explicitly requires comparing non-numerical magnitudes (e.g., duration or frequency), as the original space has proven useful to identify numerosity biases on area judgements in 5-year-old children (Tomlinson et al., 2020). Indeed, a significant influence of numerosity has been reported not only on spatial judgments of density and area involving parallel presentation of visual stimuli (Cicchini et al., 2016), but also on judgments on temporal features such as the duration of sequences of flashing dots (Dormal et al., 2006).

Our results also revealed that numerosity comparison was affected, to some extent, by task-irrelevant characteristics of the stimuli, mostly related to the temporal spread of the events. These results are in line with previous reports of temporal biases in numerosity discrimination, although the direction of the influence is inconsistent across studies (Lambrechts et al., 2013; Martin et al., 2017; Philippi et al., 2008; Tokita & Ishiguchi, 2011). Some reported an overestimation of the numerosity of dynamically appearing arrays presented in shorter time intervals, suggesting an effect of the rate of evidence accumulation on the noisiness of magnitude representations (Lambrechts et al., 2013; Martin et al., 2017). In contrast to this interpretation, we found a tendency to overestimate the number of events in longer sequences with longer blank intervals, in both visual and auditory modalities. At least one study reports an underestimation of sequences when events were separated by shorter intervals in visual, auditory, and tactile modality, with a larger underestimation of visual events, compared to other modalities, for shorter intervals (Philippi et al., 2008). However, the authors suggest that this interaction could have emerged from a flicker-fusion illusion for visual sequences, due to the high presentation rates of their stimuli (up to roughly 33 Hz) (Levinson, 1968). To avoid a similar effect, in our stimuli we kept the minimum interval between pulses above 50 ms and we used irregular durations of events and intervals. Moreover, although we cannot completely exclude that underestimation of faster sequences could have emerged from a perceptual fusion of extremely close pulses in the visual modality, the parallel result in the auditory modality, where the temporal resolution is largely below the average frequency of presentation, is a strong indicator that our results cannot be explained by a possible fusion of close events. It should also be noted that our stimulus space was designed in terms of physical, not perceptual, units. Although some studies highlighted similarities in the discrimination precision of numerical and temporal magnitudes (Feigenson, 2007; Droit-Volet et al., 2008), some studies report the opposite (Odic, 2018). Therefore, future studies could better investigate whether the intensity range in each dimension allows for a similar discriminability across all axes of the stimulus space and additionally calibrate the ranges in terms of “just noticeable difference” units.

The current design does not allow us to determine whether the non-numerical bias originated from a shared neural representation of magnitude (Bueti & Walsh, 2009) or a non-perceptual competition at the response selection level. To address this question, a similar manipulation of features could also be used to assess the spontaneous saliency of different numerical and temporal magnitudes during development (Roitman et al., 2007) or test the existence of cross-dimensional adaptation or serial dependency effects on temporal numerosity (Togoli, Fedele, et al., 2021a; Tsouli et al., 2019). Furthermore, a promising avenue for future research would be to use this type of stimuli to probe computational models of numerosity perception, which often exhibit the same type of non-numerical biases observed in human participants (Testolin et al., 2020a; Zorzi & Testolin, 2018).

The discrimination accuracy and the overall contribution of temporal magnitudes were similar in the visual and auditory sensory modalities. Although the moderate statistical evidence does not allow us to draw strong conclusions, this finding is in line with previous reports of correlations in the estimation precision of sequences of flashes and sounds, and similar numerical acuity across auditory or tactile modalities (Anobile et al., 2016; Tokita & Ishiguchi, 2016). Collectively, these results suggest that basic numerosity processing in different senses could rely on a common mechanism. It could be noted that the duration coefficient was significantly different from zero in the visual but not in the auditory modality. However, the direct contrast between the two duration coefficients was not significant. Nevertheless, we believe that a within-subject design would be more appropriate for a fine-grained comparison of coefficient values across sensory modalities.

Overall, these results illustrate how the proposed approach can be used to study numerosity perception and temporal biases in different modalities such as visual, auditory, or tactile. Crucially, this modality-nonspecific manipulation paves the way to the investigation of the impact of temporal cues in cross-modal or multimodal experimental designs as well. For this reason, we believe that it can represent a useful method to further clarify the interplay between number and time and lead to a more comprehensive understanding of human quantitative reasoning.

### Supplementary information

Below is the link to the electronic supplementary material.

Supplementary file1 (PDF 347 KB)

## Data Availability

The datasets analyzed in the current study and the code to perform the analysis are available in the GitHub repository: https://github.com/CCNL-UniPD/temporal-bias-numseq

## Footnotes

1 https://github.com/CCNL-UniPD/temporal-bias-numseq

## Code availability

The code to generate stimuli with the procedure illustrated in this study is available in the GitHub repository: https://github.com/CCNL-UniPD/temporal-bias-numseq

## Declarations

### Open practices statement

The data and materials for all experiments are available at https://github.com/CCNL-UniPD/temporal-bias-numseq and none of the experiments was preregistered.
