# Supplementary material for: Measuring temporal bias in sequential numerosity comparison
Source: Behav Res Methods. 2024 May 15;56(7):7561–73. doi: 10.3758/s13428-024-02436-x (PMC11362239; doi:10.3758/s13428-024-02436-x)
Supplement: Supplementary file 1 — Supplementary file1 (PDF 347 KB) [file 13428_2024_2436_MOESM1_ESM.pdf]

Supplementary Information

for

Measuring temporal bias in sequential numerosity comparison

Serena Dolfi [1], Alberto Testolin [2, 3], Simone Cutini [1], Marco Zorzi [2, 4]

[1] Department of Developmental Psychology and Socialization, University of Padova, Padova, Italy

[2] Department of General Psychology, University of Padova, Padova, Italy

[3] Department of Mathematics, University of Padova, Padova, Italy

[4] IRCCS San Camillo Hospital, Venice, Italy

Code pipeline description

We provide MATLAB code that uses our framework to generate a set of sequential numerical stimuli varying independently in Numerosity, Duration and Temporal Spacing, available in the Github repository: https://github.com/CCNL-UniPD/temporal-bias-numseq.

Based on selected levels of Numerosity, Duration and Temporal Spacing, the main script generates a set of sequences from all the possible combinations of these three features. Following the equations reported in the previous paragraph, each stimulus is created with a secondary function (see Figure S1), starting from the number of events (n) in the sequence and its Total event duration (TED) and Total stimulus duration (TSD), specified in frames. Sequences are created as a structure with two fields corresponding to a vector of individual event durations and a vector of inter-event intervals.

```
>> n = 7;
>> ted = 30;
>> tsd = 120;
>> event_method = 'Fixed';
>> interval_method = 'Sum';

>> [seq] = seq_stim_creator(n,ted,tsd,event_method,interval_method)

>> seq =

  struct with fields:

    ied_vec: [4 4 4 4 4 4 4]
    int_vec: [23 5 7 11 16 4 24]
```

Figure S1. Secondary function to generate one sequence. The function seq_stim_creator takes as input parameters the number of events (n), the total event duration (ted), the total stimulus duration (tsd), and the intended variability in individual event duration (event_method) and individual interval duration (interval_method) to return a structure array that defines the sequence in terms of durations in frames.

Both regular and irregular sequences can be created modifying the corresponding parameters referring to events or intervals: '*Fixed*' for homogeneous individual durations and '*Sum*' for heterogeneous individual durations, obtained through an iterative process. The output sequence is defined by two vectors:

- *ied_vec*, containing n individual event durations in frames.
- *int_vec*, containing n-1 individual interval durations in frames.

Through the main script, users can save the stimuli in a spreadsheet containing, for each stimulus, the timestamps of events and intervals and the stimulus features (see Figure S2). Moreover, the script allows the visualization of the intended and real features of the stimulus set.

name	ied_vec	int_vec	n	ted	ied	tsd	mep	dur	tmp_sp
Stim1	2 4 4 6 5 6 3	16 12 8 12 5 10 9	7	30	4.29	102	14.57	128.57	1486.29
Stim2	6 7 2 8 10 5 5	9 10 9 5 7 5 14	7	43	6.14	102	14.57	264.14	1486.29
Stim3	11 13 4 7 6 12 6	3 3 8 10 10 5 4	7	59	8.43	102	14.57	497.29	1486.29
Stim4	2 3 3 3 4 2 5 2 2 4 4 4 2 3	12 6 7 9 9 3 9 10 10 4 8 3 4 8	14	43	3.07	145	10.36	132.07	1501.79
Stim5	6 4 3 5 4 7 6 4 2 7 3 4 3 2	9 3 6 7 3 10 10 6 7 3 3 6 5 7	14	60	4.29	145	10.36	257.14	1501.79
Stim6	6 3 5 8 5 9 2 8 5 6 7 10 3 6	3 4 4 4 7 4 3 3 6 4 4 4 7 5	14	83	5.93	145	10.36	492.07	1501.79
Stim7	2 2 2 2 3 2 2 3 2 2 3 2 2 2 2 2 2 2 2 2 2 2 2 3 2 2 2 2	4 4 3 3 6 3 3 7 5 7 9 3 7 4 3 3 3 8 8 8 7 4 8 5 6 3 5 6	28	60	2.14	205	7.32	128.57	1500.89
Stim8	3 4 3 3 2 4 4 2 3 3 3 4 2 3 3 5 2 2 2 3 5 4 3 2 2 3 3 3	6 6 3 5 4 5 6 6 4 4 3 4 3 5 4 5 5 3 5 3 3 4 3 4 3 3 5 6	28	85	3.04	205	7.32	258.04	1500.89
Stim9	2 3 7 4 4 2 5 5 5 3 5 2 4 7 5 2 6 4 2 6 5 3 7 3 3 5 4 4	4 3 3 3 4 4 3 3 3 3 3 3 3 3 3 3 3 3 3 3 4 3 3 3 3 3 3 3	28	117	4.18	205	7.32	488.89	1500.89
Stim10	2 3 6 6 2 6 5	14 16 8 7 31 11 28	7	30	4.29	145	20.71	128.57	3003.57
Stim11	2 4 8 5 10 7 7	9 14 18 3 17 12 29	7	43	6.14	145	20.71	264.14	3003.57
Stim12	3 13 13 7 7 11 5	11 9 20 18 3 8 17	7	59	8.43	145	20.71	497.29	3003.57
Stim13	2 3 3 5 5 2 2 3 3 4 3 2 3 3	12 19 16 10 6 7 8 11 3 13 14 20 6 17	14	43	3.07	205	14.64	132.07	3001.79

Figure S2. Example of output spreadsheet. Users can save a table containing complete stimulus information such as the timestamps of events and intervals (in frames), number of events and continuous features (in frames).

The manipulation of duration in frames allows the generation of sequential numerical stimuli in different sensory modalities from the same timestamps. However, this can make the sequences dependent on the system used, especially for visual stimuli. The example provided and the default values used in all the scripts assume the correspondence: 1 frame = 0.01667 s (for a screen refresh rate of 60 Hz). Values of Duration and Temporal Spacing in the main script, as well as minimum IED or Interval values used in the secondary function should therefore be changed according to the system, screen refresh rate and the intended use of the output sequences.

An example on how to generate auditory stimuli (as *.wav* files) from the output timestamps (assuming that 1 frame = 0.01667 s) is provided in an additional script. In the script, users can easily modify event tone, sound sampling frequency, and amplitude, as well as visually inspect the generated audio signal. Alternatively, the stimulus set spreadsheet can be imported in the preferred software or tool for running experiments, to create stimuli on the fly from timestamps.

Psychophysical study

Power analysis

The sample size and the number of trials were selected based on a power analysis conducted using Monte Carlo simulations. To estimate the sample size required to detect a non-numerical bias within a group and to detect a difference in non-numerical bias between two groups, we extracted 1000 samples, for several sample sizes and trial numbers, from a population with mean numerical acuity and variability based on a pilot study with 5 participants performing a sequential numerosity comparison task in visual modality on 120 trials, for which we estimated a mean w of 0.35 (SD = 0.05). For each sample, we simulated individual trial-by-trial responses in the current comparison task from a psychophysical model of numerosity discrimination for half participants (G1) and from a psychophysical model of total stimulus duration for the other half (G2).

We then estimated the individual parameters of the described GLM with binomial distribution and probit link function with the log of Numerosity, Duration, and Temporal Spacing as regressors.

We can consider the power of detecting a non-numerical bias from a given sample size and a certain number of randomly selected stimuli, as the proportion of samples where we could individuate a significant Temporal Spacing coefficient in G2. Based on this procedure we estimated a minimum sample size of 20 participants and 90 trials to achieve a power above 0.90 to detect a significant non-numerical bias. The power of detecting a difference in strategy between the two groups was instead defined as the proportion of samples where we could individuate a significant difference between groups in the numerosity coefficient and the Temporal Spacing coefficient. We estimated a minimum sample size of 20 participants per group and 90 trials to achieve a power above 0.90 to detect a group difference in non-numerical bias.

To estimate the sample size required to detect a difference in numerical acuity between two groups, we extracted 1000 samples, for several sample sizes and trial numbers, from two populations characterized by different numerical acuity of $w = 0.35$ ($SD = 0.05$) and $w = 0.40$ ($SD = 0.05$), with a standardized difference between groups below the effect size reported in previous studies that found a significant difference in numerical acuity in response to visual or auditory sequences (Tokita et al., 2013). Following the same procedure as the previous simulation, the power of detecting a difference in numerical acuity between the two groups was defined as the proportion of samples, for a given sample size and a certain number of randomly selected stimuli, where we could individuate a significant difference between groups in the numerosity coefficient. We estimated a minimum sample size of 40 participants per group and 120 trials to achieve a power above 0.7 to detect a group difference in acuity with Cohen's $d = 1$.

Generalized linear mixed model analysis

To confirm the results from individual GLM fit and provide a better visualization of group results, we also fit a generalized linear mixed model (GLMM) with binomial distribution and probit link function, with the log-ratios of Numerosity, Duration, and Temporal Spacing of the first and second sequence as fixed effects and including by-subject random intercepts and slopes of Numerosity, Duration, and Temporal Spacing log-ratios. Coherently with the analyses on individual parameters, in the visual task, we found a significant β_{Num} (M (SE) = 1.73 (0.06), $t = 26.82$, $p < .001$), β_{Dur} (M (SE) = -0.14 (0.04), $t = -3.20$, $p = .001$), and β_{TmSp} (M (SE) = 0.22 (0.05), $t = 4.18$, $p < .001$). In the auditory task, we found a significant effect of numerosity ratios (M (SE) = 1.75 (0.07), $t = 22.93$, $p < .001$) and Temporal Spacing ratios (M (SE) = 0.29 (0.06), $t = 4.45$, $p < .001$), while the effect of Duration was not significant (M (SE) = -0.03 (0.04), $t = -0.80$, $p = .42$).

Supplementary references

DeWind, N. K., Adams, G. K., Platt, M. L., & Brannon, E. M. (2015). Modeling the approximate number system to quantify the contribution of visual stimulus features. *Cognition*, *142*, 247–265. https://doi.org/10.1016/j.cognition.2015.05.016

Tokita, M., Ashitani, Y., & Ishiguchi, A. (2013). Is approximate numerical judgment truly modality-independent? Visual, auditory, and cross-modal comparisons. *Attention, Perception, and Psychophysics*, *75*(8), 1852–1861. https://doi.org/10.3758/s13414-013-0526-x
